# Visual Outcome of Pars Plana Vitrectomy for Retained Lens Fragments after Phacoemulsification

**DOI:** 10.4103/0974-9233.51984

**Published:** 2008

**Authors:** Abdulrahman M. Al-Amri

**Affiliations:** From Vitreoretinal Division, King Khaled Eye Specialist Hospital, Riyadh, Saudi Arabia

**Keywords:** phacoemulsification, retained lens fragments, dropped nucleus, pars plana vitrectomy

## Abstract

**Purpose:**

To evaluate the visual outcome of patients who underwent pars plana vitrectomy (PPV) for posteriorly dislocated lens fragments after phacoemulsification.

**Methods:**

A retrospective chart review was conducted of all patients who had PPV for posteriorly dislocated lens fragments after phacoemulsification between January 2000 and September 2004 in the Vitreoretinal Service at King Khaled Eye Specialist Hospital, Riyadh, Saudi Arabia. Demographics, preexisting eye diseases, details of the previous cataract surgery, findings at presentation, details of the vitreoretinal procedures, final visual acuity, and complications observed during the follow-up were evaluated.

**Results:**

Of the 60 patients identified, 37 patients (37 eyes) had a full set of data and were included in the study. In 21 eyes (56.8%), PPV was performed within 1 week of cataract extraction; in 15 eyes (40.5%), PPV was performed more than 1 week postcataract extraction. An initial visual acuity of 20/200 or worse was found in 34 eyes (91.9%). The final visual acuity was 20/40 or better in 10 eyes (27%), and 20/200 or worse in 13 eyes (35.1%). Retinal detachments were found in 3 eyes (8.1%): 1 before and 2 after vitrectomy.

**Conclusion:**

There was no statistically significant difference in outcome between those having vitrectomy the first week after cataract surgery and those having it later; however, there was a trend of better visual outcome in early vitrectomy patients (within 1 week).

Phacoemulsification has become the preferred surgical method for cataract extraction. One of its complications is posterior capsule rupture, which usually occurs during the surgeon's learning phase. It can, however, occur with experienced surgeons. It may result in the dislocation of lens fragments into the vitreous cavity, which can lead to intraocular inflammation, cystoid macular edema (CME), uveitis, increased intraocular pressure (IOP), corneal edema, and decreased visual acuity. Pars plana vitrectomy (PPV) has been used to retrieve posteriorly dislocated lens fragments and restore vision.^1^–^10^ However, the timing of PPV and factors determining the final visual outcome remain unclear. A final visual acuity of 20/40 or better has been achieved in 51.8% to 82% of patients having the procedure.^1^,^2^ Several retrospective studies failed to show a significant difference in the outcome whether the PPV was immediately performed or delayed.^1^,^3^,^4^,^11^,^15^,^16^ The current study evaluated the visual outcome in patients undergoing PPV to manage posteriorly dislocated lens fragments following cataract extraction.

## Patients and Methods

After approval was obtained from the human ethics committee, the medical records of all patients who had undergone PPV for posteriorly dislocated lens fragments after phacoemulsification between January 2000 and September 2004 at King Khaled Eye Specialist Hospital were retrospectively reviewed. The parameters of the study included demographics, preexisting eye diseases, details of the previous cataract surgery (including date), performance of anterior vitrectomy, and intraocular lens (IOL) implantation. In addition, visual acuity using Snellen charts, IOP, anterior and posterior segment findings at presentation (including the presence of corneal edema, anterior uveitis, lens matter, vitreous and/or fibrin in the anterior chamber, hyphema, and hypopyon), the approximate size of nuclear fragments in the vitreous, vitritis, vitreous hemorrhage, retinal detachment, CME, and choroidal effusion were evaluated. Other variables studied were the details of the procedure (including date), the interval between cataract extraction and vitreoretinal surgery, use of phacofragmentation (fragmatome) and/or heavy liquids, operative complications such as retinal tear or retinal detachment, final visual acuity, and complications recorded during the follow-up.

Visual acuity was measured with Snellen charts. A poor visual outcome was defined as a visual acuity worse than 20/40. An IOP value was considered high when it was 30 mm Hg in cases with no history of glaucoma, with or without maximal medical therapy. Anterior uveitis was recorded when an anterior chamber reaction of +2 was present. The interval between cataract surgery and PPV was divided into 2 groups: within 1 week and more than 1 week. A minimum of 3 months of follow-up data after vitrectomy was necessary for inclusion in the study. Patients with less than 3 months of follow-up data were excluded.

Statistical analysis was performed using the chi-square test.

## Results

Of the 60 patients (60 eyes) identified, 37 patients (37 eyes) met the inclusion criteria and were included in the study. There were 23 men (62.2%) and 14 women (37.8%), with a mean age of 60.2 years (range, 40-85 years). The mean follow-up was 12.1 months (range, 3 months to 3 years). Of the 37 patients, 34 patients (91.9%) had an initial visual acuity of 20/200 or worse, 21 patients (56.8%) had uveitis, 16 patients (43.2%) had corneal edema, 15 patients (40.5%) had vitritis, and 1 patient had macula-off rhegmatogenous retinal detachment. The clinical features are summarized in Table 1.

**Table 1:** Clinical Features of Patients with Retained Lens Fragments on Initial Examination

Findings at Presentation	Patients, No. (%)
Visual acuity 20/200	34 (31.9)

Corneal edema	16 (39)

Uveitis	21 (77.8)

Hyphema	14 (37.8)

Fibrin in AC	2 (5.4)

Hypopyon	0 (0)

Lens matter in AC	5 (13.5)

Vitreous in AC	2 (5.4)

Vitritis	15 (40.5)

High IOP	10/36 (27.8)

Vitreous hemorrhage	5 (13.5)

Retinal detachment	1 (2.7)

Cystoid macular edema	4 (10.8)

IOP = intraocular pressure; AC = anterior chamber

Clinically significant macular edema (5.4%), Ischemic maculopathy (2.7%), and glaucoma (5.4%) were the preexisting eye diseases detected (Table 2).

**Table 2:** Preexisting Eye Diseases

Preexisting Eye Diseases	No of Eyes	%
CSME	2	5.4

Ischemic maculopathy	1	2.7

Glaucoma	2	5.4

Normal	32	86.5

Total	37	100

CSME = clinically significant macular edema

Anterior vitrectomy was performed in 30 eyes (81.1%) at the time of cataract surgery. In addition, 33 patients (89.2%) had posterior chamber IOL (PCIOL) placement, 6 patients (16.2%) were left aphakic, and 2 patients (5.4%) had anterior chamber IOL (ACIOL) placement. At the time of PPV, 1 patient had secondary IOL (PCIOL), and 1 patient underwent IOL removal because of IOL subluxation. After the second retinal reattachment surgery, 1 patient underwent IOL removal because of IOL subluxation. The time interval between the date of cataract surgery and the date of PPV ranged from 0 to 122 days. Within 1 week of cataract extraction, 23 eyes (62.2%) underwent surgery, and 16 eyes (43.2%) had a vitrectomy more than 1 week after cataract surgery. In 1 eye (2.7%), a vitrectomy was performed immediately after identification of dislocated lens fragments during the same surgical session.

All 37 patients underwent a standard three-port PPV to remove the retained lens fragments. The pars plana phacofragmentation approach was used to remove nuclear fragments in 33 patients (89.2%); the vitreous cutter was used in 8 patients (21.6%). Perfluorocarbon liquids (PFCLs) were used to float the lens fragments anteriorly for removal in 25 patients (67.6%). High IOP (30 mm Hg or more) readings were measured in 10 patients (27%): It normalized without medication after vitrectomy in 7 patients (70%), whereas the remaining 3 patients continued to require antiglaucoma medications; however, none had filtering surgery. The time interval between cataract surgery and vitrectomy among these 3 patients was within 1 week in 1 patient and more than 1 week in 2 patients.

New rhegmatogenous retinal detachment macula on occurred in 2 patients between 2 and 4 months after vitrectomy. In 1 patient (following PFCL injection, air-fluid exchange, cryoretinopexy, injection of gas, and endolaser photocoagulation), retinal reattachment and a best corrected visual acuity (BCVA) of 20/100 were achieved. The other patient (following scleral buckle, transcleral cryotherapy, air-fluid exchange, and injection of gas) achieved a BCVA of 20/50 but developed recurrent retinal detachment 1 year later. When this patient subsequently underwent scleral buckle removal, cryotherapy air-fluid exchange, endolaser photocoagulation, and removal of the subluxated IOL, his BCVA was 20/200.

The visual acuity generally improved after vitrectomy (Table 3). Before vitrectomy, the majority of patients presented with a visual acuity of 20/200 or worse (91.9%). None of these patients had a visual acuity of 20/40 or better.

**Table 3:** Visual Acuity Before and After Vitrectomy

Visual Acuity (n = 37)	Before Vitrectomy*		After Vitrectomy	
	
	No.	%	No.	%
20/40	0	0	10	27.0

20/50 to 20/100	4	10.8	14	37.8

20/200	32	86.5	13	35.1

Total	36	-	37	-

* One eye was excluded because pars plana vitrectomy was performed at the time of cataract extraction; n = number of eyes

The timing of vitrectomy compared with the visual acuity outcome is shown in Table 4. The interval between cataract surgery and vitrectomy was evaluated. Of the 21 eyes that underwent PPV within 1 week of cataract extraction, the final visual acuity was 20/40 or better in 7 eyes (33.3%). Of the 15 eyes that underwent PPV more than 1 week postcataract surgery, 3 (20%) achieved a visual acuity of 20/40 or better while 12 eyes (81.3%) had a visual acuity worse than 20/40. Anterior vitrectomy was not performed in 11 eyes (29.7%) at the time of cataract surgery. Of these 11 eyes, 6 eyes (54.5%) had a visual acuity worse than 20/40, and 3 patients had retinal tears during PPV; however, none of these patients developed a retinal detachment. Perfluorocarbon liquids were used in 14 eyes; only 3 of these eyes (21.4%) achieved a visual acuity of 20/40 or better. Therefore, PFCLs did not appear to be a protective factor against poor visual outcome (*P* = 0.3385).

**Table 4:** Interval between Cataract Extraction and Vitrectomy in Eyes with Retained Lens Fragments

Final VA(n=37)	Vitrectomy Interval	
	
	Within 1 week, n = 21* No.(%)	More than 1 week, n = 15 No. (%)
20/40	7 (33.3)	3 (20)

20/20 to 20/100	9 (42.9)	4 (26.7)

20/200	5 (23.8)	8 (53.3)

Total	21	15

* One eye was excluded because pars plana vitrectomy was performed at the time of cataract extraction; VA = visual acuity; n = number of eyes

The final visual acuity was 20/40 or better in 27% (10/37) of patients and 20/200 or worse in 35.1% (13/37). There was no statistically significant difference in outcome between patients having PPV within the first week after cataract surgery and those having it later (*P* = 0.190, Fisher's exact test); however, there was a trend of better visual outcome in early vitrectomy patients (within 1 week).

## Discussion

With the advent of phacoemulsification as the technique of choice for many cataract surgeons, there has been an apparent increase in the occurrence of retained lens fragments after cataract extraction. The exact incidence of dislocated lens fragments is rather difficult to estimate. Many cases of posteriorly dislocated lens fragments may go undiagnosed, especially when they are well tolerated by the eye with little or no symptoms. In other instances, the lens fragments may stimulate a mild inflammatory reaction, which is easily controlled medically.

Although PPV is considered the preferred surgical procedure in the management of posteriorly dislocated lens fragments following cataract surgery, a review of the literature indicates a lack of consensus regarding what effect the timing of this procedure has on the final visual outcome. According to several studies, patients undergoing PPV to manage retained lens fragments experienced a higher incidence of long-term complications (e.g., corneal edema, uveitis, glaucoma, and retinal detachment) when the performance of this procedure was delayed.^11^ In contrast, other studies found that the timing of PPV in managing patients with posteriorly dislocated lens fragments after cataract surgery did not have an effect on the final visual outcome and the rate of complications.^10^,^14^,^15^ Some have suggested that delaying vitrectomy for 2 or more weeks can facilitate the eventual procedure by allowing for softening of nuclear material, posterior vitreous detachment, and better control of intraocular inflammation and pressure.^13^

Al-Khaier and coauthors^17^ found that a delay of vitrectomy for more than 4 weeks correlated with poor visual outcome. Other large series have failed to demonstrate a statistically significant association between the timing of vitrectomy and chronic glaucoma or visual outcome.^1^,^14^

Whereas early vitrectomy is desirable, converting a complicated cataract surgery into a PPV during the same session may not be ideal. Lengthening an already prolonged case can be difficult for the patient. Achieving adequate anesthesia can also become a problem, particularly in the modern era of topical anesthesia.

In the current series, delaying vitrectomy for more than 1 week was associated with a higher percentage of patients with poor visual outcome, whereas a higher percentage of patients who underwent vitrectomy within 1 week achieved a visual acuity of 20/40 or better. However, this difference was not considered statistically significant.

Margherio et al. observed significant differences in final visual acuities based on lens status. Those with PCIOL had better vision than those with ACIOL, who had better vision than aphakic patients. The authors attributed the findings to the degree of complications encountered at the time of cataract surgery, for which lens status would be a marker.^11^ Other studies have failed to demonstrate an association between type of lens implant (i.e., anterior or posterior chamber) and visual outcome.^1^,^14^

Borne et al. observed a trend toward higher rates of retinal detachment among patients in whom phacofragmentation was used.^14^ Similarly, Al-Khaier and coauthors found higher retinal detachment rates in patients who had phacofragmentation.^17^ The use of posterior segment ultrasound in their series significantly correlated with poor visual outcome. However, other large series have not confirmed these observations.^1^,^11^ The use of PFCLs in cases of posteriorly dislocated crystalline lenses has been advocated as a means of protecting the retina against ultrasonic energy and mechanical trauma from lens fragments during emulsification.^12^ However, in the retrospective study by Borne et al., PFCL appeared to offer no clear benefit in terms of visual outcome.^14^

In a large series, Moore et al. reported retinal detachment in 19 (5.5%) of 343 patients after PPV for retained lens fragments.^18^ Ho and Zaman found retinal detachment in 4.9% of patients.^19^ In our series, retinal detachment was reported in 5.4% of patients after PPV.

We recommend the following techniques for removing posteriorly dislocated lens fragments. The remaining vitreous attachments to anterior chamber structures, the IOL, and the iris must be removed. Initially, the vitrector can be used to remove small pieces of cortex and softer pieces of nucleus following the removal of all vitreous surrounding the lens fragments. The vitrectomy should be extended peripherally using scleral depression to remove all fragments that may have become embedded in the vitreous base. This step is essential in order to avoid vitreous traction during phacofragmentation. The phacofragmentation probe is used to aspirate the lens material from the retinal surface and elevate it into the mid-vitreous, where it is emulsified. Fragmentation should be kept to a minimum so as to avoid retinal trauma. Although the posterior dislocation of lens material is a serious complication of phacoemulsification, appropriate intraoperative and postoperative management of this complication can result in good visual outcome. The main limitation of this study is the relatively small sample size. In conclusion, there was no statistically significant difference in outcome between patients undergoing vitrectomy within the first week after cataract surgery and those undergoing it later.
